# A randomized controlled phase II trial of vaccination with lysate-loaded, mature dendritic cells integrated into standard radiochemotherapy of newly diagnosed glioblastoma (GlioVax): study protocol for a randomized controlled trial

**DOI:** 10.1186/s13063-018-2659-7

**Published:** 2018-05-25

**Authors:** Marion Rapp, Oliver M. Grauer, Marcel Kamp, Natalie Sevens, Nikola Zotz, Michael Sabel, Rüdiger V. Sorg

**Affiliations:** 1 Department of Neurosurgery, Heinrich Heine University Hospital, Moorenstr. 5, 40225 Düsseldorf, Germany; 2 Department of Neurology, University Hospital Münster, Albert-Schweitzer-Campus 1, 48149 Münster, Germany; 3 Coordination Center for Clinical Trials, Heinrich Heine University Hospital, Moorenstr. 5, 40225 Düsseldorf, Germany; 4 Institute for Transplantation Diagnostics and Cell Therapeutics, Heinrich Heine University Hospital, Moorenstr. 5, 40225 Düsseldorf, Germany; 5 Department of Neurosurgery, Heinrich Heine University Hospital Düsseldorf, Moorenstr. 5, 40225 Düsseldorf, Germany

**Keywords:** Glioblastoma, Dendritic cells, Immunotherapy, Vaccination, Radiochemotherapy

## Abstract

**Background:**

Despite the combination of surgical resection, radio- and chemotherapy, median survival of glioblastoma multiforme (GBM) patients only slightly increased in the last years. Disease recurrence is definite with no effective therapy existing after tumor removal. Dendritic cell (DC) vaccination is a promising active immunotherapeutic approach. There is clear evidence that it is feasible, results in immunological anti-tumoral responses, and appears to be beneficial for survival and quality of life of GBM patients. Moreover, combining it with the standard therapy of GBM may allow exploiting synergies between the treatment modalities. In this randomized controlled trial, we seek to confirm these promising initial results.

**Methods:**

One hundred and thirty-six newly diagnosed, isocitrate dehydrogenase wildtype GBM patients will be randomly allocated (1:1 ratio, stratified by O6-methylguanine-DNA-methyltransferase promotor methylation status) after near-complete resection in a multicenter, prospective phase II trial into two groups: (1) patients receiving the current therapeutic “gold standard” of radio/temozolomide chemotherapy and (2) patients receiving DC vaccination as an add-on to the standard therapy. A recruitment period of 30 months is anticipated; follow-up will be 2 years. The primary objective of the study is to compare overall survival (OS) between the two groups. Secondary objectives are comparing progression-free survival (PFS) and 6-, 12- and 24-month OS and PFS rates, the safety profile, overall and neurological performance and quality of life.

**Discussion:**

Until now, close to 500 GBM patients have been treated with DC vaccination in clinical trials or on a compassionate-use basis. Results have been encouraging, but cannot provide robust evidence of clinical efficacy because studies have been non-controlled or patient numbers have been low. Therefore, a prospective, randomized phase II trial with a sufficiently large number of patients is now mandatory for clear evidence regarding the impact of DC vaccination on PFS and OS in GBM.

**Trial registration:**

Protocol code: GlioVax, date of registration: 17. February 2017.

Trial identifier: EudraCT-Number 2017–000304-14.

German Registry for Clinical Studies, ID: DRKS00013248 (approved primary register in the WHO network) and at ClinicalTrials.gov, ID: NCT03395587. Registered on 11 March 2017.

**Electronic supplementary material:**

The online version of this article (10.1186/s13063-018-2659-7) contains supplementary material, which is available to authorized users.

## Background

Glioblastoma multiforme (GBM) is the most frequent and aggressive malignant primary brain tumor. The yearly incidence is 3–4 per 100,000 adults [1]. The established therapeutic standard in the first-line therapy for GBM combines maximal safe resection, fractionated radiotherapy with concomitant alkylating temozolomide (TMZ) chemotherapy followed by adjuvant TMZ. This multimodal approach has improved the survival of patients significantly. Nevertheless, the prognosis of newly diagnosed GBM patients is still poor. Median overall survival (OS) is only 14.6 months, the 2-year survival rate is 27.2%, and disease recurrence is universal, with virtually all patients dying within 1.5 years after relapse [2, 3]. Thus, there is a clear need for novel therapeutic modalities.

Dendritic cell (DC)-based, active anti-tumoral cellular immunotherapy aims at generating autologous cytotoxic effector T cells, which kill tumor cells specifically. Because of their central role in initiating such T-cell responses, DC are used as cellular adjuvant for application of tumor antigens in tumor vaccines [4, 5]: Patients are vaccinated with tumor antigen-loaded DC, with the concept that they will migrate to local lymph nodes, present tumor antigen-derived peptides on human leukocyte antigens (HLA) and initiate an anti-tumoral T-cell response.

DC-based vaccination therapy was first applied in a clinical trial in 1996 for B-cell lymphoma [6], but only in 2006 has the clinical efficacy of this approach been proven in a phase III trial for hormone-refractory prostate cancer patients [7]. For GBM, since the first case report in 2000 [8], 38 studies and case reports have been published, initially applying DC vaccination in recurrent disease, but more recently as part of first-line therapy in newly diagnosed patients as well [9–46]. Close to 500 GBM patients have been treated with DC vaccination in these studies, with the age of patients ranging from 1 to 80 years.

Previous studies have documented feasibility and safety. There have been no reports of severe side effects (≥ grade 3) attributable to vaccination, except for one patient with gross residual tumor after surgery, who suffered from peritumoral edema, which was controllable by glucocorticoids [16, 23]. Frequently observed mild and easily controllable toxicities (≤ grade 2), which may be attributable to DC vaccination, have been injection site reactions with itching, pain, erythema, induration and lymph node swelling as well as flu-like symptoms, fever, fatigue, myalgia, headache, edema and meningeal irritation. Thus, overall toxicity of DC vaccination therapy is limited, and vaccination has also no severe impact on the quality of life of patients. Indeed, the quality of life of patients has been reported to remain stable during vaccination therapy [27, 28, 42] or even to improve [35, 37, 44].

Induction of antigenic, target-directed immune responses have been observed in the course of vaccination, with detection of interferon (IFN)-γ responses being most informative [25], but anti-tumoral cytotoxic responses [10, 17–19] and an increase in tetramer positive cytotoxic T cells [18, 31, 36, 44, 45] have been reported as well. Several studies identified immunological responders based on delayed-type hypersensitivity-like reactions [20], IFN-γ responses [20, 25, 30, 39, 43, 45, 46] or cytotoxic responses [19] and reported longer survival times of responders [19, 20, 25, 30, 39]. Moreover, long-term survivors with durable IFN-γ responses have been identified [43]. Such associations of anti-tumoral immunity with survival are in line with the proposed mechanism of action of vaccination therapy and strong arguments for the efficacy of this type of active immunotherapy in GBM.

Prolonged survival of vaccinated patients has been reported compared to matched or historic controls [10, 18–20, 26, 29, 32, 40, 43]. In a recent meta-analysis of 9 studies, Cao et al. [47] concluded that vaccination significantly improves OS (1–5 years) as well as PFS rates (1–4 years), e.g., OS rates at 4 years were 20% and 1%, at 5 years 14% and 0% for vaccinated and non-vaccinated patients, respectively.

For newly diagnosed GBM, in a recent randomized phase II trial with 34 patients [35], the OS rate at 24 months (44.4%) as well as the median OS (31.9 months) of the vaccination group compared favorably to the control group (18.8%; 15.0 months). In another controlled study including 25 patients, median OS (17.0 vs. 10.0 months) as well as OS rates at 2 years (7.7% vs. 0%) compared favorably for the vaccinated patients [37]. In 16 non-randomized studies, the median OS of newly diagnosed GBM patients ranged from 11.0 to 38.4 months [10, 17, 19, 24, 26, 28–30, 32, 34, 38–40, 43, 45, 46], with one [10] of the three studies [10, 24, 29] reporting the lowest median OS (11.0–15.0 months), nevertheless describing an improved OS compared to matched controls (15.0 vs. 8.4 months).

Thus, results of previous DC vaccination trials have been encouraging, although they cannot yet provide robust evidence of clinical efficacy. Moreover, DC vaccination can be easily integrated into the current first-line therapy, and there is a rational for such an integration besides retaining a therapy with proven clinical efficacy for the patients; it may allow exploiting synergies between the treatment modalities: (1) after resection/radio-/chemotherapy, patients are in a state of minimal residual disease, which is probably beneficial for immunotherapy because of the lower tumor load and the depletion of immunosuppressive cells [19, 23], (2) TMZ appears to improve immunological responsiveness [48–50], probably by reducing regulatory T cells (Treg) [50, 51] and interfering with their recruitment to the tumor [52], (3) the recovering lymphocyte compartment after chemotherapy appears to be beneficial for induction of anti-tumoral responses [53], (4) dying tumor cells after radiochemotherapy result in local pro-inflammatory conditions and a release of tumor antigens, which could improve homing of tumor-specific effector T cells to the brain tumor [54] and (5) there is an increased responsiveness to TMZ chemotherapy after DC vaccination [17]. Therefore, it now appears mandatory to determine efficacy of DC vaccination in a randomized, controlled trial on a large cohort of newly diagnosed GBM patients with standard therapy as comparator. If the hypothesis is proven correct that DC vaccination as add-on to the standard therapy in GBM significantly prolongs survival with acceptable toxicity and without significantly reducing the quality of life of patients, this trial will contribute to its implementation as therapy in newly diagnosed GBM. Otherwise, clarification of efficacy is also urgently needed for counseling GBM patients, who are aware of the apparently promising results of this therapeutic approach.

## Methods

### Study design

This is a national, prospective, multicenter, open-label, randomized phase II study with two parallel groups, with the participating hospitals being located in the state of North Rhine-Westphalia in the proximity of Düsseldorf (a list of participating centers can be obtained from the sponsor or the registry). In the active recruitment period of 30 months, 136 patients with newly diagnosed, isocitrate dehydrogenase (IDH) wildtype, primary GBM [55] will be randomized at a 1:1 ratio after near-complete resection and successful production of tumor lysate. For a patient to be enrolled, all eligibility criteria (see below) must be met and free vaccine production capacity has to be available (enrollment may have to be paused). Enrollment of a patient is initiated by an investigator submitting by fax the Randomization Request Form to the Coordination Center for Clinical Trials, which will perform the randomization and inform the investigator of the outcome and allocation of the patient to the control or the vaccination arm of the study. The randomization list has been generated by the Coordination Center for Clinical Trials, using randomly selected block sizes of 4, 6, 8 and 12 patients. It will be centrally applied with concealment by the Coordination Center for Clinical Trials. The assignment to the two groups will be stratified according to O6-methylguanine-DNA-methyltransferase promotor methylation status, because it is an independent prognostic factor in TMZ chemotherapy [2, 56].

All patients will receive the current standard of care with proven clinical efficacy [2, 3, 57] for newly diagnosed GBM consisting of (1) FGS, (2) fractionated RT (60 Gy, 2 Gy/day, 5/7 days, 6 weeks) with concurrent TMZ chemotherapy (75 mg/m^2^/day, 6 weeks) and (3) subsequent adjuvant TMZ chemotherapy (150–200 mg/m^2^/day, days 1–5 of 28-day cycle, six cycles). TMZ dose reduction due to toxicity follows standard rules: Concomitant TMZ chemotherapy will be interrupted when the absolute neutrophil count is 500–1500/μl or the platelet count is 10,000–100,000/μl or non-hematological toxicity grade 2 occurs. Concomitant TMZ chemotherapy will be discontinued when the absolute neutrophil count is < 500/μl or the platelet count is < 10,000/μl or non-hematological toxicity grade 3 or 4 occurs. Adjuvant TMZ chemotherapy dose will be reduced when the absolute neutrophil count is < 1000/μl or the platelet count is < 50,000/μl or non-hematological toxicity grade 3 occurs. Adjuvant TMZ chemotherapy will be discontinued when hematological and non-hematological toxicities persist despite the lowest dose of TMZ (75 mg/m^2^/day).

**Fig. 1:**
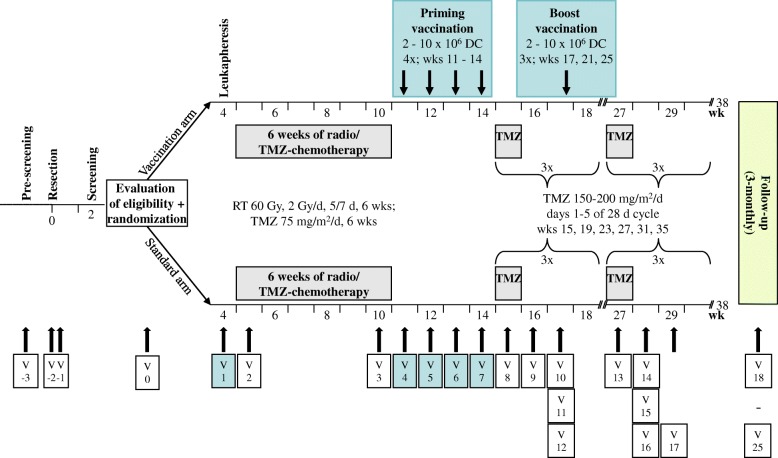
GlioVax study design. After resection, patients are randomized 1:1 into the vaccination and standard arms of the study. Patients in the standard arm are treated with fractionated radiotherapy (RT) and concomitant temozolomide (TMZ) chemotherapy, followed by 6 cycles of adjuvant TMZ. Patients in the vaccination arm receive 4 priming vaccinations and 3 boosting vaccinations with tumor-lysate-loaded, mature DC as add-on therapy to the standard therapy, between radiochemotherapy and adjuvant TMZ and in the first 3 cycles of adjuvant TMZ, respectively. The overall intervention period in both arms is 38 weeks, follow-up is 2 years. After screening (visits − 3 to − 1), 18 study visits (visit 0 (baseline) to visit 17) and 8 3-monthly follow-up visits are planned, to assess safety, efficacy and quality of life. Visits indicated in blue are in the vaccination arm only. Other visits are for patients in both arms of the study

In the control group, patients receive this standard treatment with proven clinical efficacy (comparator). Patients in the vaccination arm of the study will receive as add-on up to seven tumor lysate-loaded, mature DC vaccines (2–10 × 10^6^ cells each applied by intradermal injection): four priming vaccines in weekly intervals during the period between concomitant radiochemotherapy and adjuvant chemotherapy and three monthly boosting vaccines in the third week of the first three 28-day cycles of adjuvant TMZ (Fig. 1). Availability of less than three vaccines is regarded as treatment failure. Vaccines will be applied irrespective of whether concomitant TMZ has to be terminated or adjuvant TMZ has to be reduced or terminated due to toxicity. Corticosteroids should be avoided or kept to a minimum 1 week before until 1 week after vaccination. In case of temporary need for high-dose corticosteroids 1 week before vaccination (> 8 mg/day dexamethasone or equivalent), vaccination has to be postponed. Any other anti-cancer treatment, including agents with only suspected anti-cancer effects, any other investigational or experimental treatment, any other active immunotherapy or any treatment that interferes with immune function, including persistent immunosuppressive therapy (e.g., for rheumatism) should be avoided or kept to a minimum during the treatment period. If a patient misses two or more of the priming vaccinations, this is regarded as treatment failure. If one priming vaccination or one, two or all three boost vaccinations are missed, it will be attempted to add the missed vaccinations (maximum: 3) as additional boost vaccinations, always in the third week of subsequent adjuvant TMZ cycles (cycles 4–6).

**Fig. 2:**
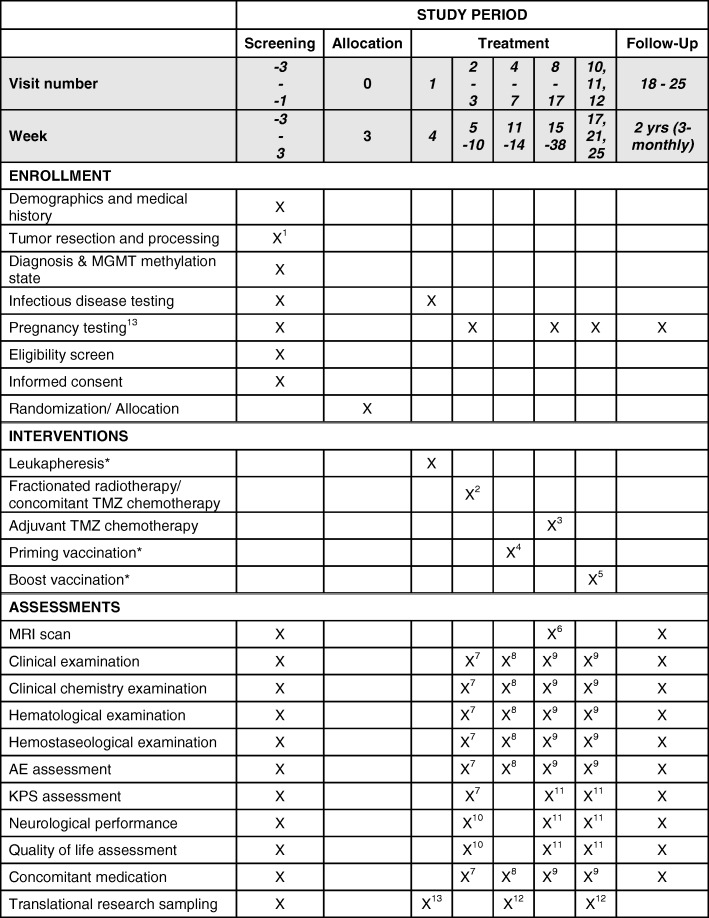
Visits for enrollment, treatment and assessments. After screening for eligibility and allocation (visits − 3 to − 1), 18 study visits (visits 0–17) are planned for treatment and assessments during the treatment period, followed by 8 additional visits (visits 18–25) during the 2-year follow-up. ^1^Resection is performed in week 0 (visit − 2); ^2^weeks 5–10: 6 cycles of concomitant radiochemotherapy; ^3^weeks 15, 19, 23, 27, 31, 35): 6 cycles (days 1–5 of 28-day cycle) of adjuvant temozolomide (TMZ) chemotherapy; ^4^weeks 11–14: 4 priming vaccinations; ^5^weeks 17, 21, 25: 3 boost vaccinations; ^6^weeks 16, 28, 37: magnetic resonance imaging (MRI) scans; ^7^weeks 5, 10: clinical, clinical chemistry, hematological, hemostaseological examinations and adverse events (AE), concomitant medication and KPS assessments; ^8^weeks 11–14: clinical, clinical chemistry, hematological, hemostaseological examinations and AE and concomitant medication assessments; ^9^weeks 15, 17, 21, 25, 29, 33, 38: clinical, clinical chemistry, hematological, hemostaseological examinations and AE and concomitant medication assessments; ^10^week 10: neurological performance and quality of life assessments; ^11^weeks 15, 29, 38: KPS, neurological performance and quality of life assessments; ^12^tumor tissue and blood for translational research studies are sampled during resection, leukapheresis and at the 7 time points of vaccination; ^13^pregnancy tests are performed at monthly intervals until 6 months after the treatment period; *vaccination arm only

Patients are informed verbally and on the basis of the informed consent form in-depth about the importance of vaccination being performed in line with the protocol. They receive the contact information of the coordinating investigator and are invited to contact him in case of any questions. Standard therapy will be performed in all participating hospitals. Production and application of vaccines will be in Düsseldorf only. The intervention period is 38 weeks, follow-up is 24 months. Overall, a duration of 63 months (first patient in to last patient out 54 months) is anticipated for the trial (Fig. 2).

A formal interim efficacy analysis will be performed when approximately 50% of the expected 87 events have occurred in the treatment and control groups combined. At the interim, a group-sequential methodology will be applied to the analysis. The O’Brien-Fleming approach [58] will be used and overwhelming efficacy will be concluded if the two-sided *p* value of the log-rank test for the difference in median OS between treatments is < 0.003. The decision to close the trial based on overwhelming or inferior efficacy of vaccination will be taken by the sponsor together with the coordinating investigator after critically assessing ethical and safety aspects.

### Eligibility criteria

All inclusion and exclusion criteria (Table 1) will be evaluated by the local investigators (neurosurgeons and/or neuro-oncologists) and in case of residual tumor volume after surgery and diagnosis/tumor cell content of tumor sample confirmed by central neuroradiologist and neuropathologist, respectively.

**Table 1:** Inclusion and exclusion criteria

Key inclusion criteria	Key exclusion criteria
Newly diagnosed, monofocal GBM, isocitrate dehydrogenase wildtype (WHO grade IV; [57]) histologically confirmed by central neuropathologist	Medical history of severe acute or chronic disease with poor prognosis, autoimmune disorder, immunodeficiency, organ allograft or prior malignancy (≤ 3 years)
Near-complete resection (≤ 5 ml residual tumor volume) confirmed by central neuroradiologist on magnetic resonance imaging (MRI) scan within 72 h postoperative	Infection with human immunodeficiency virus, hepatitis B virus, hepatitis C virus or *Treponema pallidum* or other severe infection requiring hospitalization or i.v. antibiotics or anti-viral treatment (≤ 2 weeks)
Patients ≥ 18 years of age	Known allergy or intolerability to TMZ or any component of the capsules, dacarbazine, the contrast agent or the DC vaccine
Karnofsky performance status ≥ 70%	History of bleeding diathesis or coagulopathy
Sterile tumor sample of ≥ 150 mg with tumor cell frequency ≥ 60%, as determined by central neuropathologist, available for vaccine production	Preexisting myelosuppression
Successful production of sterile, avital tumor lysate	Previous radiotherapy to head and neck
Systemic corticosteroids tapered down to ≤ 2 mg of dexamethasone or equivalent per day within 7 days postoperative	Previous (≤ 6 weeks. or ≤ 5 half-lives) treatment with specific immunostimulatory agent
Adequate hepatic, renal, liver and bone marrow function and blood coagulation	Previous (≤ 4 weeks) treatment with live, attenuated vaccine
Use of highly effective contraception	Treatment of GBM in another clinical trial with therapeutic intervention or current use of any investigational agent
Signed informed consent	Known pregnancy or breast-feeding
	O6-methylguanine-DNA-methyltransferase promoter methylation status equivocal

Legend: *DC* dendritic cell, *GBM* glioblastoma multiforme, *i.v.* intravenous, *TMX* temozolomide, *WHO* World Health Organization

### Study endpoints

The primary objective of the study is to determine whether survival of newly diagnosed GBM patients treated with lysate-loaded, mature DC vaccines as add-on to the standard of care is superior to the treatment with the standard of care alone. The primary efficacy endpoint is OS measured from the day of surgery until death. Secondary objectives are comparing (1) progression-free survival (PFS), (2) 6-, 12- and 24-month OS and PFS rates, (3) the safety profile, (4) overall and neurological performance and (5) the quality of life between the two treatment groups.

Following the baseline visit, 17 visits during the treatment period of 38 weeks and 8 visits during the 2-year follow-up period have been planned to assess primary and secondary endpoints. Study visits have been scheduled as close as possible to those of the standard treatment to limit any additional burden for the patients. Tumor progression will be assessed by magnetic resonance imaging (MRI) according to modified RANO (response assessment in neuro-oncology) criteria [59, 60]. Following standard protocol, MRI examinations after the 72-h post-surgical MRI scan will start in week 16 (6 weeks after completion of radiochemotherapy) and will be performed thereafter in 3-monthly intervals during the treatment as well as in the follow-up periods.

At each visit (for visit plan see Fig. 1), clinical examinations as well as laboratory assessments will be performed to monitor safety, with toxicity coded according to the Medical Dictionary for Regulatory Activities (MedDRA) and graded according to the National Cancer Institute (NCI) common terminology criteria for adverse events 4.03 (CTCAE 4.03). All adverse events (AE) occurring in the course of the trial will be documented. Each AE is rated by the investigator whether a correlation with the trial drug can be assumed (adverse drug reaction; ADR) or not. All severe adverse events (SAE), irrespective of whether associated with the trial drug (severe adverse drug reaction; SADR) or not and whether rated by the coordinating investigator as unexpected or a suspected unexpected severe drug reaction (SUSAR) will be reported to the sponsor within 24 h, and every SUSAR will be reported by the sponsor to the National Competent Authority and the Ethics Committee. Pregnancies are documented on a separate Pregnancy Report Form and reported to the sponsor immediately after learning of the pregnancy.

The Karnofsky performance status, neurological performance status (Minimal Mental State Examination-2; [61]), quality of life (EORTC QLQ-C30 and QLQ-BN20 questionnaires; [62, 63]) and psycho-oncological distress (Distress Thermometer [64] and Hospital Anxiety and Depression Scale [65] will be assessed.

Patients withdrawn from the trial prematurely by the investigator or at their own request are asked to participate in the Termination Visit (clinical, clinical chemistry, hematological, hemostaseological examinations, MRI, AE assessment, overall and neurological performance, quality of life) and to the 3-monthly follow-up according to study protocol until the end of the follow-up period. At a minimum, all AE should be followed until they are fully and permanently resolved or have stabilized. If the patient withdraws or declines consent for disclosure of future information, no further evaluations will be performed, and no additional data will be collected, but the use of any data collected before of the withdrawal will be retained, to be used further as may be necessary, e.g., to assess efficacy and safety.

Safety and efficacy data will also be assessed by an independent Data Safety and Monitoring Committee (DSMC) composed of a neurosurgeon, a neuro-oncologist, a neuropathologist and a biostatistician, who had no involvement in the design of the study, are not involved in its conduct other than through their role on the DSMC, and who have no financial interest in the outcome of the study. Assessments will be based on semiannually safety reports and the interim report. The DSMC may recommend to the sponsor at any time during the trial discontinuation of the trial or modification of the protocol for safety reasons. It may also recommend to stop the trial in case of insufficient enrollment or overwhelming/inferior efficacy. The DSMC Charter provides a detailed description of the responsibilities and activities of the DSMC.

### Data management, monitoring and auditing

All data will be collected in electronic case report forms (eCRF). The eCRF will be implemented in a Good Clinical Practice (GCP)-compliant clinical data management system with electronic data capture functionality, which provides the capability to perform the major data management activities within a consistent, auditable and integrated electronic environment (query management, data entry, data validation). Range, validity and consistency checks will be implanted in the eCRF for application during data entry. All data entry, modification or deletion will be recorded automatically in an audit trail indicating the original value, the new value, the reason for change, who made the change and when the change was made. A digital signature is implemented and included in the audit trail. Periodically, central monitoring of the data is performed and actions are taken to ensure data quality (queries, re-training). Implementation of the eCRF has been performed by data managers in cooperation with biostatisticians from the Coordination Center for Clinical Trials. All procedures are documented in the protocol and in the Data Management Manual.

Source data remain in the respective hospital information system. Source documents, in particular documents with plain-text names of the participating patients, are kept under strict control of the investigator at the trial site. They will not be transferred to the sponsor or other persons that are not authorized by the patient in the data protection declaration part of the informed consent form. Data recorded in the eCRF are pseudonymized; the Patient Identification List is kept under strict control at the individual trial sites.

Periodic monitoring of the trial will be performed on site in the trial centers by clinical research associates to ensure the safety of the trial participants, the trial itself as well as the correctness of the collected data. They will check the availability of the patient’s informed consent, adherence to eligibility criteria, adherence to treatment according to the trial protocol, safety parameters and completeness of the trial documents in the trial center. The monitor confirms completed source data verification by electronic signature in the eCRF. Monitoring is described in detail in the Monitoring Manual. The on-site trial monitoring is combined with central monitoring with regular data checks.

To ensure sponsor oversight, audits will be performed by an independent auditor at the sponsor’s request. Especially, the vaccine production and its documentation as well as the correct delivery of the tumor and blood samples will be audited. In case of non-compliance of the study centers an audit can also be performed.

### Advanced therapy medicinal product: DC vaccine

The DC vaccines for the GlioVax trial are produced specifically for each patient according to established standard operating procedures with production permission by the local authorities. The vaccines consist of two autologous cellular components: (1) tumor lysates as a whole-cell source of tumor antigens and (2) monocyte-derived, mature DC.

Lysates are produced from autologous tumor material of the patients obtained from fluorescein-guided surgery (FGS). FGS is an established standard surgical procedure for GBM [57]. Because the “solid” part of the tumor can be identified intraoperatively, high-purity tumor material can be obtained, and only samples with a tumor cell content of at least 60% are used for vaccine production. After resection, tumor material is stored frozen at − 80 °C until processing, which involves mechanical dissection and repeated freeze/thaw cycles to generate the lysate.

DC are generated from monocytes enriched immunomagnetically from non-mobilized leukapheresis products. A two-step serum-free culture system is used, to successively generate immature and mature DC [66, 67]. Monocytes are cultured for 6 days in Teflon bags in the presence of granulocyte-macrophage-colony-stimulating factor and interleukin-4 to generate immature DC, which are loaded with tumor antigens by adding the autologous tumor lysate to the cultures. Maturation of DC is induced during an additional 3-day culture period, using the classical pro-inflammatory maturation cocktail described by Jonuleit et al. [68], containing tumor necrosis factor α, interleukin-1β, interleukin-6 and prostaglandin E2, which allows generation of CD83+, fully mature DC with high purity and potent T-cell stimulatory and T_H_1-polarizing activity. After ex-vivo generation of the tumor-lysate-loaded, mature DC, they are stored frozen until the day of application, on which they are thawed, washed, resuspended in 0.9% sodium chloride solution and transferred into a 1-ml syringe for immediate application.

### Sample size considerations and statistical analysis

The sample size calculation is based on the primary outcome OS. A median OS of 14.6 m has been reported for the control intervention [2, 3]. In eight recent smaller phase I/II trials on newly diagnosed GBM treated with lysate-loaded DC as add-on to radiochemotherapy, median OS of 35.9 months [32], 34.4 months [17], 31.9 months (control group 15.0 months) [35], 30.5 months [45], 28.0 months [30], 27.4 months [38], 24.0 months [28], 18.4 months [34] and 17.0 months (control group 10.5 months) [37] have been reported. Based on a critical appraisal of these data, taken into consideration potential treatment dilution effects for the results from the smaller phase I/II trials, we have assumed a prolongation of OS of 12 months for the vaccination group (OS 26.6 months). When the sample size in each group is 61, with a total number of events required of 87, a 0.050-level two-sided log-rank test for equality of survival curves will have 80% power to detect the difference between a median OS in the control group of 14.6 months vs. 26.6 months in the test group (hazard ratio for death, 0.55).

In order to compensate for dropouts (i.e. patients dropping out prior to 24 months without having an event) the total sample size is planned at 136 patients (i.e. an approximately 10% dropout rate). Mainly due to a tumor content of samples for vaccine production below 60% or unsterile tumor material (7/37 (19%); determined at the Institute for Transplantation Diagnostics and Cell Therapeutics, Düsseldorf) or residual tumor volume above 5 ml upon radiological evaluation (17%; [57]), it is anticipated that approximately 35% of GBM patients will fail to meet inclusion/exclusion criteria after resection. Therefore, 210 patients have to be screened to allow for enrollment of 136 patients in the trial.

Each year, about 450–500 patients with newly diagnosed GBM are treated in the participating centers, of which about 280 (60%) would fulfill the inclusion/exclusion criteria of the trial. It is anticipated that about 260 of these patients would agree to participate and that it would be possible to recruit 210 patients in the 30-month recruitment period. Thus, recruitment of 136 patients seems feasible. The recruitment rate would be about one patient/center/month and centers would have to assess one to two/patients/month for eligibility.

Evaluation of results will be based on the Statistical Analysis Plan. Efficacy is measured by OS computed from the day of surgery until event or censored at last follow-up according to the Kaplan-Meier method with two-sided log-rank statistics for comparison. Primary efficacy analysis will be based on the intention-to-treat population; patients lost to follow-up are censored at the time of last contact. If required, a model-based Bayesian analysis for imputation of missing data will be used, and missing values imputed using the posterior predictive distribution of the model. In addition, a sensitivity analysis based on the per-protocol population will be performed.

All patients will be evaluated for safety; assessment of safety will be based on descriptive and explorative methods. For explorative analyses of secondary endpoints, classical statistical tests will be applied (e.g., log-rank test for survival data (PFS), chi-square/Fischer’s exact test for categorical data and *t* test/Mann-Whitney *U* test for continuous data). In addition, 95% confidence intervals will be calculated when applicable.

A Cox regression model will be used for explorative analysis of overall survival and progression-free survival with the covariates (1) treatment group, (2) recursive partitioning analysis classes I–IV as defined by Mirimanoff et al. [69] and de Vleeschouwer et al. [70], which already have been documented to influence outcome of standard as well as of vaccination therapy, the prognostic markers, (3) MGMT promotor methylation status, (4) extent of resection (complete vs. near-complete), (5) age, (6) center, (7) dexamethasone medication and (8) the therapeutic intervention in case of tumor progression. Subgroups of vaccinated patients will also be defined based on their immunological responsiveness as well as immunosuppressive scores (see below).

## Discussion

### Trial design

The trial design attempts to minimize parameters, which have been identified to affect clinical and immunological outcome, in order to generate informative results on efficacy of DC vaccination in GBM. Only adults with confirmed newly diagnosed primary GBM after near-complete resection will be included, with all patients undergoing standard radio/TMZ chemotherapy. Moreover, DC vaccination with high-purity, tumor-lysate-loaded, mature DC will be fully integrated into standard therapy.

Randomization will be the main method against bias. DC vaccines are produced specifically for the individual patient, using the patient’s own tumor and leukocytes as starting material. We have considered it to be unethical to subject the patients of the control group to the potential risks and the stress of leukapheresis merely for blinding purposes. Thus, only patients of the vaccination group are subjected to leukapheresis and, therefore, there will apparently be different procedures applied to patients in the control and vaccination groups, which also requires different logistics. Therefore, blinding the study has not been possible.

### Tumor-induced immunosuppression

After activation by the DC, effector T cells can cross the blood-brain barrier and exert their effector function in the brain [71], with a contribution of luminal antigen presentation by endothelial cells to identify the specific site for effector T cells to enter the brain parenchyma [54] and a final tuning of T-cell effector functions by the brain microenvironment [71, 72]. Thus, the basic pre-requisites for DC vaccination therapy of GBM are in place, and numerous animal studies could confirm that it is a a promising therapeutic approach in brain tumors [73–77]. However, there is also tumor-induced immunosuppression, and several contributing mechanisms have been identified [78, 79], including an increase in regulatory T cells in the blood as well as in the tumor microenvironment, which is associated with impaired T-cell responsiveness [80]. Such immunosuppression may hamper DC vaccination therapy and, therefore, has to be overcome, to improve or even allow efficacy. In GBM, it correlates with the size of the tumor, and surgical cytoreduction can restore T-cell responsiveness, at least partially [81–84]. Certain chemotherapeutics, including TMZ, which is used in the standard treatment of GBM, have also been reported to reduce T_reg_ in the periphery as well as in the tumor [50–52], which might be important to increase the vaccine efficacy [85, 86]. Thus, there is a rationale to maximally reduce the tumor burden, prior to DC vaccination therapy and combine it with TMZ chemotherapy.

### Lysate as a source of tumor antigens

For the GlioVax trial, autologous tumor lysates are used as a whole-tumor cell source of tumor antigens. Even when high-purity tumor tissue is collected by FSG, there is an abundance of normal self-proteins in tumor lysates. Nevertheless, induction of autoimmunity or other severe side effects attributable to the use of lysates for vaccine production have not been reported. Therefore, lysates can be considered a safe source of tumor antigens.

Lysates most likely will contain multiple tumor antigens, which are present in the individual tumor of a patient (including the various cellular subsets within the tumor), i.e. the patient’s full antigenic repertoire, ensuring antigenic diversity, thereby reducing the risk of escape of tumor-antigen-loss variants [87]. Since the respective proteins are endogenously processed in the DC, presentation on HLA-class I and II molecules is possible and independent of the HLA type of the patient, thereby allowing induction of cytotoxic as well as T_H-_responses at the same time, which is a pre-requisite for the development of an efficient cytotoxic T-cell response. Furthermore, lysate may provide in addition to the tumor antigens the necessary signals for guiding effector T cells to the brain [88]. Whether lysates are superior to molecularly defined tumor antigens in inducing anti-tumoral immune responses or more beneficial clinically in GBM is currently unknown. However, Neller et al. concluded from the analysis of 173 published immunotherapy trials on various tumor entities, including melanoma, renal cell and hepatocellular carcinomas, lung, prostate, breast, colorectal, cervical, pancreatic and ovarian cancers, a higher objective response rate (8.1% vs. 3.6%) when whole-tumor-cell antigens compared to molecularly defined tumor antigens were used [89]. Therefore, lysates represent an immunologically potent source of tumor antigens.

### Maturation state of DC

In the GlioVax trial, mature DC rather than immature or semi-mature DC are used as vaccine cells, because (1) they have potent T-cell stimulatory activity [67], (2) they polarize responses towards T-helper cell (T_H_1) [67], which is required for efficient induction of effector cytotoxic T cells [90], (3) they express CC-motive chemokine receptor type 7 [67]), which is required for lymph node homing [91, 92], (4) they are phenotypically stable upon withdrawal of cytokines [67], (5) they are resistant against immunosuppressive cytokines like interleukin(IL)-10 or transforming growth factor(TGF)-β2, which may be produced by the tumors [93, 94] and (6) only immature and semi-mature DC, but not fully mature DC have been implicated in tolerance induction [95, 96], which would be detrimental to the intended induction of anti-tumoral immune responses. Indeed, Yamanaka et al., who used mature as well as immature DC in their study, reported a trend towards a better outcome in GBM patients vaccinated with the mature DC [20] and de Vries and colleagues reported that tumor-antigen-loaded, mature DC are superior to immature DC in the induction of immunological responses in melanoma patients [97]. Moreover, Dhodapkar et al. showed a decline of the influenza-matrix-peptide-specific T-cell response in healthy individuals after vaccination with matrix-peptide-loaded, immature DC and urge caution with the use of immature DC to enhance tumor immunity [98].

### Route of vaccine administration

Depending on the route of application, DC can be detected in different organs. Intravenous application results in a rapid enrichment in liver, lung and kidney, but is highest in spleen, whereas after subcutaneous application, there is a marked accumulation of DC in the draining lymph nodes, with a preferential paracortical localization in the T-cell areas [99]. When intradermal application is used instead, even more DC reach the T-cell areas of lymph nodes in mice [100] as well as in humans [101], with only mature, but not immature DC, efficiently migrating to the lymph nodes in melanoma patients [102], probably due to their expression of the CC-motive chemokine receptor 7, which is missing on immature DC [91, 92]. DC can be detected already after 30 min in the lymph nodes; they are at a maximum after 48 h, and they appear to persist for up to 14 days [103–105]. Although only 4% of injected DC may reach the lymph nodes, this appears to be sufficient for effective induction of anti-tumoral immune responses [106, 107]. Due to these advantages, intradermal application of DC vaccines is used in the GlioVax trial.

### MRI and pseudoprogression

Standard radiochemotherapy of GBM may result in a transient increase in contrast enhancement in a large fraction of patients, which will subside eventually without any change in therapy [59]. Therefore, early MRI diagnostics at week 10 after completion of radiochemotherapy is not feasible. Similarly, immunotherapy may result in inflammatory reactions, which mimic tumor progression and may even include the appearance of “new lesions” on MRI scans [60]. This pseudoprogression, irrespective of whether due to standard radiochemotherapy or immunotherapy, is difficult to differentiate from true tumor progression. Moreover, immunotherapy may result in delayed responses, i.e. patients may still benefit from therapy although showing signs of progressive disease initially [60]. To minimize premature declaration of tumor progression and discontinuation of therapy, in the situation of suspected progression, thus when patients meet the radiological RANO criteria for progression initially, patients may continue study medication until progression has been confirmed on a subsequent MRI scan performed within 4–6 weeks [59]. However, there still may be uncertainty regarding progression. Due to the sometimes long-lasting nature of particularly immune-related effects, distinguishing progression from pseudoprogression unequivocally can require a prolonged (≥ 3 months) observation period as summarized by Okada et al. [60]. Therefore, subsequent confirmatory MRI scans in 4–6-week intervals will be performed and patients can continue study medication as long as the clinical and neurological status of the patient has not been worsening substantially and the study medication is well tolerated, does not interfere with therapeutic interventions for imminent complications, and the investigator assesses an overall possible clinical benefit [60]. Re-operation will be considered, to allow for final histological confirmation of tumor progression [59, 60], if the suspected lesion if surgically accessible and it is the investigator’s medical judgement that the patient may benefit from re-operation as reported previously [108, 109]. The use of additional imaging techniques (e.g., fluoroethyl-tyrosine positron-emission tomography (FET-PET)) to confirm MRI findings is also possible. The exact time of progression is defined retrospectively, when pseudoprogression and pseudoresponses can be excluded. Further treatment in case of progression is at the discretion of the investigator.

### Translational research program

GBM-mediated immunosuppression may interfere with the development of potent anti-tumor immune responses and, thus, could be inversely correlated with clinical and immunological outcome of DC vaccination [79, 110, 111]. Moreover, detection of induction of anti-tumoral immune responses after vaccination and superior clinical outcome in immunological responders compared to non-responders would be in line with the proposed mechanism of action of DC vaccination therapy and could be a strong argument to mechanistically support the efficacy of this type of active immunotherapy in GBM. Therefore, our translation research program aims to identify immunological responders and non-responders within the group of vaccinated patients and to evaluate the association between individual anti-tumor immune responses, the degree of peripheral and local immunosuppression before, during and after the vaccination procedure, and clinical outcome.

At defined time points during priming and boost vaccination, phenotypical and functional analysis of peripheral blood mononuclear cells (PBMC) will be performed. Multicolor flow cytometric analysis will focus on the assessment of the frequencies of CD4+ and CD8+ T-cell subsets including regulatory T cells, natural-killer-cell subsets and myeloid-derived suppressor cells (MDSC) and the expression of PD-1, PD-L1, CTLA-4 and CD107a [112–114]. Furthermore, interferon (INF)-γ ELISPOT and qPCR responses of peripheral blood mononuclear cells (PBMC) and multicytokine responses (IFN-γ, IL-2, IL-10, TGF-β2, IL-17) in patient serum and conditioned media of specifically restimulated cells in the course of vaccination will be assessed. In addition, we will try to establish short-term cultured tumor cell lines from individual patients that will be used as targets for GBM-specific cytotoxic responses in CD107a degranulation assays [115].

For selected HLA-A*0201- and HLA-A*2402-positive immunological responders, we will further attempt to identify the target antigen of the anti-GBM immune response. Tetramer analysis and INF-γ responses towards molecularly defined antigens (e.g., EphA2, MAGE1, HER2, TRP2, IL-13R2, EGFRvIII, survivin, CMVp65 peptides and the IMA950 targets [43, 116, 117] will be performed after stimulation of PBMC with peptide-loaded and unloaded autologous DC. In addition, we will make use of selected Peptivators® (Miltenyi Biotec, Bergisch Gadbach, Germany) in non-HLA-A*0201 or non-HLA-A*2404 patients.

Moreover, tumor tissue will be assessed by immunohistochemistry and tissue microarrays to determine local immunosuppressive factors in vaccinated patients. Analysis will include, but is not necessarily limited to, the frequency of regulatory T cells and MDSC and the expression levels of TGF-β2, IL-10, indolamin-2,3-dioxigenase, PD-L1 and arginin-1. If surgical resection is performed at the time of progression, tumor material will be reanalyzed as mentioned above to evaluate whether the composition and phenotype of the tumor microenvironment at baseline and after treatment correlate with clinical efficacy (Additional file 1).

## Trial status

Protocol version: GlioVax_Prüfplan_V02_F, 01.08.2017.

Approval by the national regulatory authority (Paul-Ehrlich Institute): #3011/01.

Patient recruitment is planned for January 2018.

## Additional file

Additional file 1: Standard Protocol Items: Recommendations for Interventional Trials (SPIRIT) 2013 Checklist: recommended items to address in a clinical trial protocol and related documents. (DOCX 63 kb)

## Abbreviations

ADR: Adverse drug reaction(s)
AE: Adverse event(s)
CTCAE: Common terminology criteria for adverse events
DC: Dendritic cell(s)
DSMC: Data Safety and Monitoring Committee
eCRF: Electronic case report form(s)
FET-PET: Fluoroethyl-tyrosine positron emission tomography
FGS: Fluorescence-guided surgery
GBM: Glioblastoma multiforme
GCP: Good Clinical Practice
HLA: Human leukocyte antigen(s)
IDH: Isocitrate dehydrogenase
IFNγ: Interferon- γ
IL-2, -10, -17: Interleukin-2, -10, -17
MDSC: Myeloid-derived suppressor cells
MedDRA: Medical Dictionary for Regulatory Activities
MRI: Magnetic resonance imaging
NCI: National Cancer Institute
OS: Overall survival
PBMC: Peripheral blood mononuclear cells
PFS: Progression-free survival
RANO: Response assessment in neuro-oncology
RT: Radiotherapy
SADR: Severe adverse drug reaction(s)
SAE: Severe adverse event(s)
SUSAR: Suspected unexpected severe drug reaction(s)
TGF-β2: Transforming growth factor-β2
T_H_1: T-helper cell 1
TMZ: Temozolomide
T_reg_: Regulatory T cell(s)
